# A smart and flexible approach for aggregation of adjacent polygons to meet a minimum target area or attribute value

**DOI:** 10.1038/s41598-023-31253-z

**Published:** 2023-03-16

**Authors:** Marcello Schiavina, Michele Melchiorri, Sérgio Freire

**Affiliations:** European Commission, Joint Research Centre, Via E. Fermi 2749, 21027 Ispra, VA Italy

**Keywords:** Software, Environmental sciences, Mathematics and computing

## Abstract

Many geospatial analyses require flexible aggregation of adjacent units to meet a minimum target area or attribute value. This is usually accomplished using several non-automated and complex GIS tasks. We developed an integrated user-friendly approach and algorithm implemented in the ‘GHS-SmartDissolve’ tool, which addresses minimum mapping unit or attribute value requirements, layers resolution mismatch, spatial uncertainty or modifiable areal unit problem in GIScience. This method automatically dissolves adjacent features updating fields’ values to reach a minimum target area or attribute value, using a flexible settings framework to meet user requirements. Also provided as a toolbox for ArcGIS (Esri), the approach is demonstrated by (i) estimating the mean particulate matter concentrations for all municipalities in Italy in 2011 by combining a coarse grid of global PM2.5 concentrations with fine administrative units; (ii) estimating boundaries of Metropolitan areas in Portugal as aggregation of municipalities, by matching their total population.

## Introduction

The progress in Geographic Information Systems (GIS) applications is reflected in the continuous increase of tools and services to facilitate the development of geospatial models and analyses, especially dealing with open large geospatial datasets^1–5^. Among the topics requiring further advancements, the aspects of spatial incompatibility (e.g. mismatch between thematic and spatial resolution) and heterogeneity (e.g. minimum mapping unit, MMU; minimum attribute value, MAV; and modifiable areal unit problem, MAUP) remain significant issues in Geographic Information Science (GIScience)^6^. These issues are particularly important in spatial analysis and modelling, in particular for environmental or socioeconomic studies where vector-based data (e.g. features representing analysis units) are used to summarize the information contained into raster layers^7,8^. It frequently happens that the two or more layers involved in the analysis differ in their spatial scale or resolution (e.g. a kilometric grid combined with polygons smaller than one square kilometer), recommending the application of modelling and geospatial techniques to harmonize the spatial detail of the datasets involved^9,10^. To solve the spatial incompatibility between layers, two common approaches are usually applied: (a) refining or further disaggregating raster data (e.g. oversampling, areal weighting, or modelling interpolation^11–14^); or (b) dissolving vector data and attributes, when a hierarchical structure is available (e.g. administrative units), thus generalizing all features to a coarser spatial subdivision (e.g. from 331 Divisional Secretariat Divisions to 25 Districts in the exercise of Edirisinghe and Maduranga^15^). However, the first approach imposes assumptions (i.e. a model) over an already modelled layer that might contradict those used to produce it, or it could potentially lead to an abuse of the original spatial resolution. On the other hand, the second approach by being ‘blind’ to the specific size of features and other attributes, may cause unnecessary loss of spatial detail for the analysis and cannot be applied when an explicit hierarchical structure is missing (i.e. not encoded in the attribute table). We propose a third way as an alternative that preserves the original raster data and enables an automated *smart* aggregation of only those features presenting a spatial incompatibility for the analysis (saving time with respect to a manual processing of the layer), retaining the attribute structure and characteristics without jeopardizing the subsequent analyses. Freire et al*.*^16^ applied this methodology when combining a layer of Italian communes with a 30-arcsecond population grid. Given the small scope of the analysis, the authors manually flagged polygon features deemed incompatible with the raster layer resolution (i.e. too small), identified the best adjacent feature to be dissolved with and ran a ‘dissolve’ geoprocessing operation for their aggregation.

Moreover, numerous studies may require that analyzed units reach a certain MMU for results to be meaningful or more robust (e.g. when summarizing point data: a sufficient number of points may need to be considered to avoid the small population problem; or when upscaling from raster data to vector units whose size is smaller or similar to the raster cell size), while not losing the information stored in the units’ attribute fields. A similar constraint could be imposed on count field values by requiring a MAV to be reached (e.g. to obtain a sufficient amount of people within census enumeration or reporting units, as in Qader et al*.*^17^). In particular circumstances, highly heterogeneous areal values could lead to bias in data interpretation (e.g. when comparing densities between features which differ widely in their area), an issue known as MAUP^18^. The MAUP can be mitigated in both its zone configuration (geometry) and scale sub-problems through the aggregation of analysis units aiming at reaching more uniform areas, or by considering results from different aggregations (e.g. as computed by Stillwell et al.^19^).

Another persistent challenge is the frequent mismatch between thematic and spatial detail or scale, as in the case of features having low geometric/positional accuracy but having high thematic/attribute reliability. One example would be the resident population of the municipalities of a given region or country being known with higher certainty than the actual delineation of their individual map boundaries. Such challenge would benefit from the availability of a robust aggregation method that preserves the overall attribute values (i.e. correctly sums population, including from multipart features) while decreasing the positional uncertainty associated with smaller, specific quantities.

Aggregation of vector spatial data is typically accomplished through the ‘dissolve’ spatial operation, which has become one of the most common and useful procedures applied in GIS^20^. In available ‘dissolve’ tools, the aggregation operates on polygonal features that share the same category or code (i.e. boundaries are removed between adjacent polygons that have the same value for a specified attribute). However, in some cases this category or code is missing while other analyses require more flexible and sophisticated aggregations, such as reaching a defined threshold area (i.e. minimum surface) or a minimum value of a count attribute (e.g. minimum population size) by dissolving adjacent features. However, these dissolve procedures should correctly handle multipart features (i.e. a single vector feature composed of more than one polygon) and accurately summarize attributes. Several studies^3,7,21–25^ proposed probabilistic (i.e. Markov Chain Monte Carlo) or GIS-based generalization methods for polygons (*sensu* Johnston et al.^26^: “the appropriate representation of the two-dimensional polygon resolution”). These methods are based on clustering of polygons where the aggregation of features depends on similarity in attributes or on balances of compactness and within-area homogeneity. Some GIS vendors, such as ‘ETGeoWizard Dissolve Polygon’ (https://www.ian-ko.com/ET_GeoWizards/UserGuide/Dissolve_Polygons_Wizard.htm), include enhanced ‘dissolve’ functionalities. However, such products are commercial and the mis-handling of multipart features implies the modification of the initial spatial distribution without the preservation of total volume of all count field values. Nonetheless, the requirement of volume preservation is critical for count data in several applications (e.g. dealing with population census layers or any similar quantitative environmental measure at polygon level). This situation leaves a research and operational gap between the standard dissolve tool, which is limited to the aggregation by attribute field, and the polygon clustering methods. This gap can be narrowed by ready-to-use simple algorithms that improve the GIS operation ‘dissolve’ by automating procedures that are usually accomplished through several computer intensive but manual GIS tasks^27^. These tasks include dealing with object attributes during the dissolve process and/or setting thresholds to dissolve features having certain characteristics and until those thresholds are met.

In this article, we present the development of a new approach, implemented in the ‘GHS-SmartDissolve’ tool, to automate the aggregation of adjacent polygons in a more flexible and smarter way than standard dissolve approaches. This approach addresses several open challenges (i.e. MMU or MAV requirements, resolution mismatch between layers, spatial uncertainty or MAUP) by automatically dissolving adjacent polygonal features to reach a pre-defined minimum area or value of a target attribute field, while also updating the other attribute fields’ values. The algorithm is implemented in a MATLAB^®^ compiled command line tool (no license needed, runtime included in the installer), with an optional interface as ArcGIS toolbox, and it is freely available for download and use at https://ghsl.jrc.ec.europa.eu/tools.php. Enabling its direct use in ArcGIS software (Esri) facilitates the application within GIS projects and increases its usability by making it easily accessible to this wide user base. We illustrate the usefulness and flexibility of the approach in two types of real-world data problems: (i) aggregation of administrative units to match a scale suited for environmental analysis (resolution incompatibility and MMU problem); and (ii) aggregation of municipalities to estimate higher-level administrative units by matching population from an independent authoritative dataset (MAV problem).

## Materials and methods

To improve and automate the geospatial aggregation procedure, we developed and tested an integrated flexible approach, which has been implemented into an efficient algorithm, along with a user-friendly interface as toolbox for ArcGIS. Flexibility increases the range of options to meet different analysis requirements while efficient integration of tasks enables automated data processing. Finally, provision of this approach as a tool benefits applicability and usability. The approach and tool have been designed to enable a wide range of applications and user needs, namely to solve MMU, MAV or mitigate MAUP by aggregating adjacent features (i.e. non-overlapping polygons that share at least a border segment) to reach a target area or a minimum attribute value, setting the working options according to each specific problem.

The approach offers several customization options (described in detail later in this section) to reach the target area of attribute value for all or selected features: four methods to order features for analysis, seven criteria to guide the dissolution of adjacent polygons, and several field operations for accurately updating the attribute fields of resulting features. This work builds upon an early study^28^ that set the stage for this methodology, where we improve and extend the capabilities in order to broaden the range of potential applications (e.g. performance enhancement, increased working options, revision of adjacency algorithm). The main improvements of the ‘GHS-SmartDissolve’ tool with respect to the first version are:

- the possibility of working with any count attribute of the vector layer as target field (i.e. dissolve units by summing the values of a specific field until this reaches a given value);
- an increased variety of rules to guide the dissolve procedure;
- the possibility of using multiple criteria as dissolve rules with a lexicographic approach (only as command line);
- the possibility of selecting only a set of features to apply the target value (in this case each feature could have its own target);
- the possibility of restricting the adjacency within groups of features (using attribute field values);
- the possibility of controlling aggregations that exceed the target values by imposing a tolerance to the maximum value of the target field (or area);
- the possibility of limiting the adjacency only if the share of common boundary is greater than a given value;
- the optimization of the algorithm that more than halves the computing requirements (both in memory and computational time).

**Figure 1:**
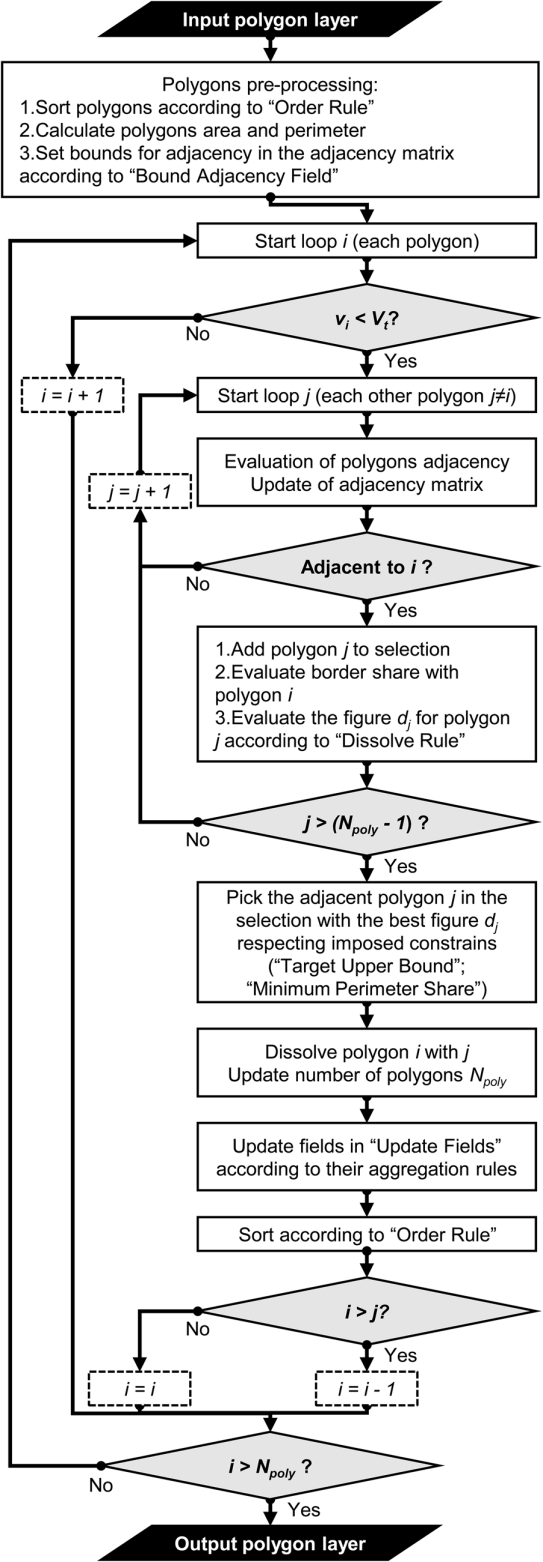
Aggregation algorithm flowchart: *i* is the external loop cursor (loop for testing target); *j* is the inner loop cursor (loop for adjacency test); *V*_*t*_ is the user-defined threshold value of target field; *v*_*i*_ is the value of target field for the current feature *i*; *d*_*j*_ is the objective value for each adjacent feature *j* to be maximized or minimized in the selection according to ‘Dissolve Rule’; *Npoly* is the total number of features.

Figure 1 shows the flowchart of the developed algorithm from the input polygon layer, which could be a shapefile or a vector layer within a Geodatabase (i.e. gdb file, only for the ArcGIS toolbox), to the final output. The objective of the processing of the vector layer is to obtain another vector layer as output having all polygons or those selected respecting a minimum size (MMU) or a minimum value of an attribute field (MAV), whenever possible (isolated features might not reach the desired threshold as they are not modified by the algorithm due to the absence of adjacent features). Therefore, the developed algorithm targets a user defined area value or field value that the polygon aggregation should reach, using two main working options: a standard approach targeting all features, and the ‘Seeds’ option, which focuses the processing on selected features used as seeds.

In the standard approach, all features can be processed according to different ordering rules (‘Order Rule’). The algorithm can follow the original order (‘ID’) or can sort the features in ascending (‘MinMax’) or descending order (‘MaxMin’) of their size or field value. While using the sorting options the user controls the processing order of the features, using the “ID” option can produce different results according to the actual polygon ordering in the vector layer. The type of ordering imposes a priority on those polygons with greater or smaller distance to the target area or field value. The alternative ‘Seeds’ setting of the ‘Order Rule’ option enables the possibility to select a specific subset of polygons that should go through the aggregation with adjacent ones and meet the target criterion (e.g. only a subgroup of polygons that needs to reach the imposed threshold on area of field value). The algorithm will place these features at the beginning of the list, according to the user list order, and will limit the loop (i.e. the external loop in Fig. 1) to only these, by setting the number of features, ‘*Npoly*’, to the number of seeds. In this setting, each seed could target different sizes or field values, but is not allowed to merge with other seeds. If needed by the subsequent computations, all features are processed for calculation of their area and perimeter. This step is performed through an internal reprojection of the layer to a Global Equal Area projection (World Mollweide, ESRI:54009), to reduce areal distortion as much as possible, and calculated with planar coordinates. Finally, the algorithm initializes an adjacency matrix identifying the potential adjacencies between polygons showing overlaps in their bounding boxes. The process of aggregation of polygons could be open or partially controlled. In the first case, no adjacency limit is imposed, while in the second case the user imposes a field to hierarchically bound the adjacency (‘Bound Adjacency Field’). The hierarchical bounding is a way to guide the aggregation among polygons sharing a characteristic and is not intended to fully limit the aggregation (i.e. if no adjacent polygon is found in the bounded set, the aggregation continues selecting an adjacent polygon among the others).

When pre-processing is completed, the algorithm cycles the features (from *i* = 1 to *i* = *Npoly*) and at each step it tests the size or field value requirements. Once it finds a feature *i* that does not satisfy the target test (i.e. *v*_*i*_ < *V*_*t*_, where *v*_*i*_ is the value of target field for the current feature *i* and *V*_*t*_ is the user-defined threshold value of target field), it searches for all adjacent features among all other features *j*. For each feature *j*, if adjacency is not yet tested nor imposed in the pre-processed adjacency matrix, the tool evaluates the adjacency with feature *i* and estimates the length of shared border by using the following developed algorithm:

1. multipart features to single-part;
2. evaluation of all intersection points between single-part polygons of the original feature *i* and single-part polygons of the original feature *j* by using the MATLAB^®^ built-in *polyxpoly* function (all polygon vertices along the shared border line are included in the intersection points list, see https://www.mathworks.com/help/map/ref/polyxpoly.html) and assignment of edge label to each of them (polygons’ vertices have double assignment to both edges they belong to);
3. calculation of shared border length by summing all lengths between intersection points for each couple of single-part polygons.

If at point 2 there are more than one intersection point on the same edge for both features, the adjacency is set to true. If at point 2 all intersection points are not a vertex of feature *i* nor of feature *j*, the algorithm highlights a geometry issue (i.e. overlap) and cannot calculate the length of shared border (adjacency is set to true, but shared border length is left equal to zero). Therefore, geometrical issues (i.e. overlaps or borders not collinear) in the input layer could affect the accuracy of the process when based on perimeter or shared segment length.

If no adjacent polygon is found (i.e. the feature is isolated and external, *sensu* Johnston et al.^26^), the algorithm proceeds to the next feature (i.e. *i* = *i* + 1); otherwise, it calculates for each adjacent polygon a performance index *d*_*j*_. The performance index *d*_*j*_, is assessed according to a rule selected by the user (‘Dissolve Rule’). The feature *j* associated to the best performance index is selected for aggregation. Seven different ‘Dissolve Rules’ are implemented in order to satisfy several user needs: (i–ii) minimum or maximum value of the field selected as target (or area) of the feature *j* (‘MinValue’, ‘MaxValue’); (iii–iv) minimum or maximum density of the field selected as target in the polygon area of feature *j* (‘MinDensity’, ‘MaxDensity’; available only when attribute field is selected as target as computed using the field value divided by the polygon area, e.g. target population field can use population density to guide the dissolve); (v) maximum length of border shared between feature *i* and feature *j* (‘MaxBorder’); (vi) minimum total perimeter of the dissolved feature merging *i* and *j* (‘MinPerimeter’); and (vii) maximum compactness as the isoperimetric quotient (i.e. the ratio of the total area over the area of the circle having the same perimeter^29^) of the dissolved feature using *i* and *j* (‘MaxCompactness’). Only for the command line tool, there is the possibility to select multiple rules. In this case the multi-criteria analysis applies a lexicographic method to select the feature to be dissolved.

The algorithm implements other two controls to the selection procedure. Whenever the target size or feature value is an optimal value to be reached and not merely a threshold to be overcome, it is possible to set a limit of the target value for the output (‘Target Upper Bound’), thus removing all polygons among those adjacent that would lead to an excessive value or size (i.e. resulting field value or area are bigger than a user defined tolerance, as percentage of the target value). When the shared boundary length is concerned, the user might impose a minimum share of perimeter for polygons to be eligible among the adjacent features (‘Minimum Perimeter Share’).

Once selected according to the best performance index, the feature *j* is merged with feature *i* and the total number of features, *Npoly*, is reduced by 1 (not in ‘Seeds’ ordering setting, as feature selected and dissolved is not accounted in the *Npoly* count). A selected list of feature attribute fields can be updated according to main standard rules available in most dissolve toolboxes (i.e. sum, min, max, mean and standard deviation for numeric fields or concatenation, first and last for textual fields). Original feature *i* is then substituted with the newly created one and feature *j* is removed from the list. With the exception of ‘Seeds’ ordering setting, the algorithm continues sorting the features according to the ‘Order Rule’ by placing the newly created feature in the respective position; it leaves *i* cursor to the same value, if *i* < *j*, or updates the cursor to the previous value (i.e. *i* = *i* − 1), if *i* > *j*. In ‘Seeds’ ordering setting, the newly created feature is placed at the end of the seed list and the cursor *i* is left at the same position (cursor will increase only when current feature satisfies its target) in order to let all seed features grow by one feature per step. This procedure has been implemented to avoid that one seed feature close to another seed loses the possibility to “grow” only because the second one was before in the list and already merged with all surrounding polygons).

Once the last feature is processed (*i* > *Npoly*) the algorithm ends and the resulting layer is saved as a shapefile or exported into the geodatabase of the input feature layer (only in ArcGIS toolbox).

**Figure 2:**
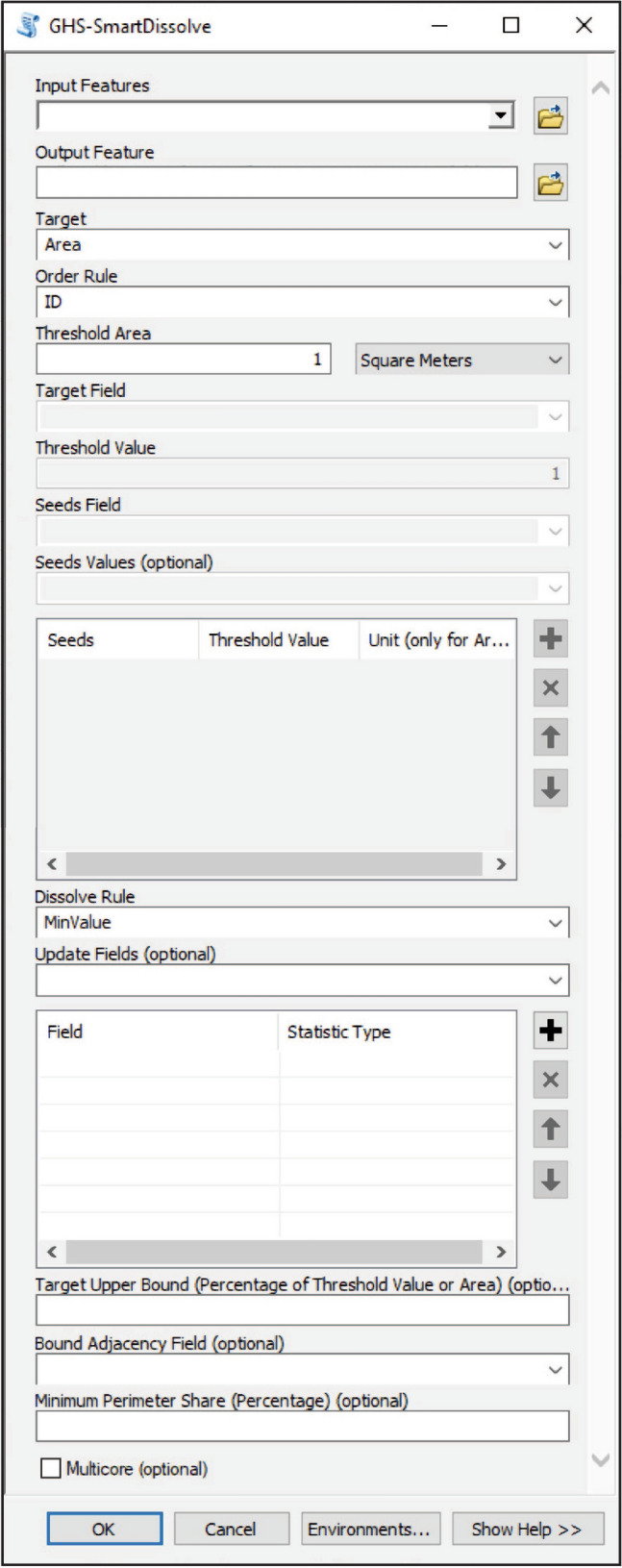
‘GHS-SmartDissolve’ ArcGIS (Esri) toolbox interface.

The tool implementing the described approach, named ‘GHS-SmartDissolve’, is freely available as a standalone command line tool at https://ghsl.jrc.ec.europa.eu/tools.php where a detailed user guide and an email contact for support are also provided. However, to increase the usability of the developed algorithm we built an ‘arcpy’ interface (Fig. 2) to use the tool as ArcGIS 10.3 + (Esri) toolbox.

## Example applications

In this section, we provide a practical demonstration of how the presented method allows the user to automatically and efficiently accomplish dissolve procedures that would otherwise require intensive and manual GIS tasks. We first illustrate the case of a resolution mismatch between two layers in performing zonal statistics to summarize the particulate matter concentration (PM2.5, a raster layer with a resolution of 0.01 degrees) in Italian municipalities (several of which not fully containing a single pixel of PM2.5 layer). We show how the tool can tackle the issue without modifying the original global PM2.5 grid and by keeping the spatial size of units as small as necessary without going to the upper administrative level. Moreover, we highlight how the tool ensures that all municipalities and respective populations in Italy are considered in analyzing PM2.5 concentrations as we test a correlation analysis between population density and these PM2.5 concentrations, a relationship suggested in other studies^30^.

Then, we present the case of estimation of boundaries for two Metropolitan Areas in Portugal (Lisbon and Porto) for which only the point locations (i.e. a latitude–longitude pair) and the respective total populations are available. Since these Metro areas are composed of municipalities, estimating their spatial extent requires aggregating adjacent municipalities until the Metro population is reached.

This second example application deals with a possible MAV problem with seeds polygons (i.e. those intersecting the point features) that require merging with adjacent ones in order to reach a threshold value of an attribute field (i.e. the population count). Without the tool here presented such results could only be obtained by manually selecting adjacent features one by one and manually checking if the population target is met.

The approach proposed in this study and implemented in the ‘GHS-SmartDissolve’ toolbox offers a solution for both problems. The first result is obtained by merging all polygons below the areal threshold with adjacent ones to match the MMU requirement and successfully completing the PM2.5 zonal statistic analysis at the selected administrative level without imposing other modelling assumptions. Moreover, the procedure preserves the total population volume and allows analysis of population density and correlation with PM2.5 taking into account all Italian population. The second result is obtained by merging the selected seeds polygons with those adjacent until they reach the required MAV in order to estimate the boundaries of the Metropolitan Areas of Portugal.

### Particulate matter concentration in Italian communes

The summarization of raster values in areas delineated by polygon features is one of the most frequent procedures in GIS-based spatial analysis. However, often this procedure requires particular attention to the relative resolutions of the two layers. As an example, we propose the analysis of PM2.5 annual concentration in all Italian municipalities in 2011, including those where their areal size causes spatial incompatibility with the raster layer to be summarized.

The freely available data on particulate matter concentration selected is the global PM2.5 grid map for 2011 (Global Annual PM2.5 Grids^31^). Among existing particulate matter layers this has the highest spatial resolution, 0.01 degrees (about 1 km at the equator). We used the Italian Statistical Institute (ISTAT) delineation of administrative boundaries of the 2011 national census^32^ as reference for Italian municipalities, joined with data on the respective population from the 2011 census available online. Such layer accounts for 8092 municipalities with areas that range from 0.12 km^2^ of Atrani, a tiny municipality on the Amalfi Coast, to the 1285.8 km^2^ of Rome. Four municipalities already have original areas smaller than the pixel size of PM2.5 layer (ranging between 0.84 and 1.01 km^2^ over the Italian territory). Moreover, the PM2.5 layer is provided at the finest resolution of the information sources incorporated, but it “does not fully resolve gradients at the gridded resolution due to influence of information sources having coarser resolutions”^31^. Therefore, any spatial analysis would benefit from an aggregation at coarser resolution, thus increasing the MMU for the analysis. Data are distributed as GeoTIFF files and are in WGS84 coordinate system (EPSG:4326).

This is a typical MMU or resolution mismatch problem, frequent when conducting local-level analyses with global layers. All municipal features below a given areal size and their respective population could be associated with unrealistic values of PM2.5, therefore biasing analyses of correlation with population density. Such bias could originate in small features and respective population not being gridded (therefore missing their respective PM2.5 value) or in the abusive upscaling (i.e. zonal statistic) of coarse PM2.5 values in small features, due to constraints of the geospatial procedure to rasterize the feature at the PM2.5 layer resolution. Previous typical solutions for such problem would involve the use of the upper administrative level (i.e. provinces), reducing the spatial detail of the analysis, or the refinement of the raster layer using advanced modelling approaches and modification of input data.

The first step to capture the PM2.5 data within each Italian municipality with a zonal statistic is to rasterize the vector layer to obtain the zones on the target grid of the raster data (i.e. PM2.5 layer). This rasterization into a 0.01 degrees grid, co-registered with the PM2.5 layer, is performed using ArcMap (ESRI) ‘Polygon to Raster’ toolbox with ‘maximum combined area’ (MCA) cell assignment. Even though the MCA algorithm, that assigns each pixel to the polygon with the maximum relative overlap, helps to mitigate problems of rasterization and resolution mismatches, it still produces zones with big discrepancies of areal representation (Table 1) with respect to the original vector layer. When rasterized, more than 15% of municipalities are represented with a difference in area exceeding 10% (versus original vector area), with differences exceeding 40% in 59 of them. Four municipalities are not even represented in the raster layer. The direct use of this raster layer in the zonal statistic to summarize the particulate matter values at municipal level could lead to biased association of PM2.5 values in some municipalities. Some features could receive the contribution of many pixels that are not representative of the area, while others would be missing in the zonal statistics table and further analyses would not take their population into account. The application of the aggregation algorithm here proposed to enforce a suitable MMU before the rasterization procedure ensures the size (scale) of aggregation zones (administrative units) is compatible with the resolution of the environmental variable under analysis (PM2.5) and allows preserving the whole population volume of Italian municipalities for the subsequent analysis. In this case, we tested the approach with a minimal set of options (Table 2): several levels of threshold areas (i.e. three MMU values), to assess the sensitivity to the gridding process, and a ‘Dissolve Rule’ to produce the most compact polygons as possible in the dissolve procedure (i.e. ‘MaxCompactness’).

**Table 1:** Sensitivity analysis of surface of municipalities to different areal thresholds in dissolve operation.

Difference (%)	Original		SD9		SD16		SD25	
0–2.5	3245	(40.1%)	3154	(46.2%)	3055	(54.7%)	2827	(63.9%)
2.5–5	1890	(23.4%)	1783	(26.1%)	1494	(26.8%)	1104	(25.0%)
5–10	1705	(21.1%)	1389	(20.4%)	895	(16.0%)	467	(10.6%)
10–20	895	(11.1%)	460	(6.7%)	132	(2.4%)	21	(0.5%)
20–40	298	(3.7%)	33	(0.5%)	4	(0.1%)	2	(0.0%)
> 40	59	(0.7%)	2	(0.0%)	2	(0.0%)	2	(0.0%)

Municipalities are grouped in four bins by differences in their area as represented in the original vector layer and in the rasterized versions (0–2.5%; 2.5–5%; 5–10%; 10–20%; 20–40% and > 40% difference). The area of municipalities rasterized at 0.01 degrees using the original vector layer (Original) is compared with layers obtained by applying the proposed aggregation algorithm and imposing different areal thresholds as MMU. Here only the most significative areal thresholds are reported: about 9 times the pixel size (3 × 3 pixels: 9 km^2^, SD9); about 16 times the pixel size (4 × 4 pixels: 16 km^2^, SD16) and about 25 times the pixel size (5 × 5 pixels: 25 km^2^, SD25).

**Table 2:** Parameter settings used for the example applications considered in this analysis.

	Target	Target field	Threshold	Order rule	Dissolve rule	Seeds field	Seeds values Seed—threshold	Update field Field—stats
*SD9*	Area	–	9 km^2^	MinMax	MaxCompactness	–		COMUNE—concatenate POP_2011—sum
*SD16*	Area	–	16 km^2^	MinMax	MaxCompactness	–		COMUNE—concatenate POP_2011—sum
*SD25*	Area	–	25 km^2^	MinMax	MaxCompactness	–		COMUNE—concatenate POP_2011—sum
*WUP*	Field	RPOP11	–	Seeds	MaxDensity	DTCC01	1312 – 1,223,600 1106 – 2,684,500	DTCC01—concatenate

The WUP application also uses the ‘*Bound Adjacency Field*’ parameter enabled and set to NUTS3 (i.e. the upper administrative level; Nomenclature of Territorial Units for Statistics, for explanation see https://ec.europa.eu/eurostat/statistics-explained/index.php?title=Glossary:Nomenclature_of_territorial_units_for_statistics_(NUTS)).

Among the several tested MMUs we report about the three most significative values. The first MMU enforced (‘SD9’) is 9 km^2^, about 9 times the PM2.5 pixel area. This MMU was selected to ensure that the final feature has an area at least comparable to a square of 3-by-3 PM2.5 pixels, so several pixels contribute to each summary in a zone. In this scenario, 1271 features are dissolved with adjacent ones, thus reducing the share of features whose difference to their raster representation is above 10%. The second tested MMU enforced (‘SD16’) is 16 km^2^, about 16 times the pixel area, comparable to a square of 4-by-4 PM2.5 pixels. With 2510 municipalities merged with adjacent ones, the representation deviation is lower with only 2.5% of features rasterized with a difference greater than 10% in area and almost 55% with a difference lower than 2.5%. The third scenario (‘SD25’) imposes an MMU of 25 km^2^, about 25 times the pixel area, for a comparable area of 5-by-5 PM2.5 pixels. By imposing this MMU, only 0.5% of features have a representation in the raster layer with a deviation greater than 10% of the original area. In all scenarios two municipalities, Ventotene and Isole Tremiti, show a significative difference (above 40%) in their area represented in the raster layer. Such municipalities are islands with no adjacent municipality to be merged with. However, by using the ‘GHS-SmartDissolve’ in all three scenarios, all municipalities and respective populations become represented in the raster layer made compatible with PM2.5 layer, contrary to using the original features.

**Figure 3:**
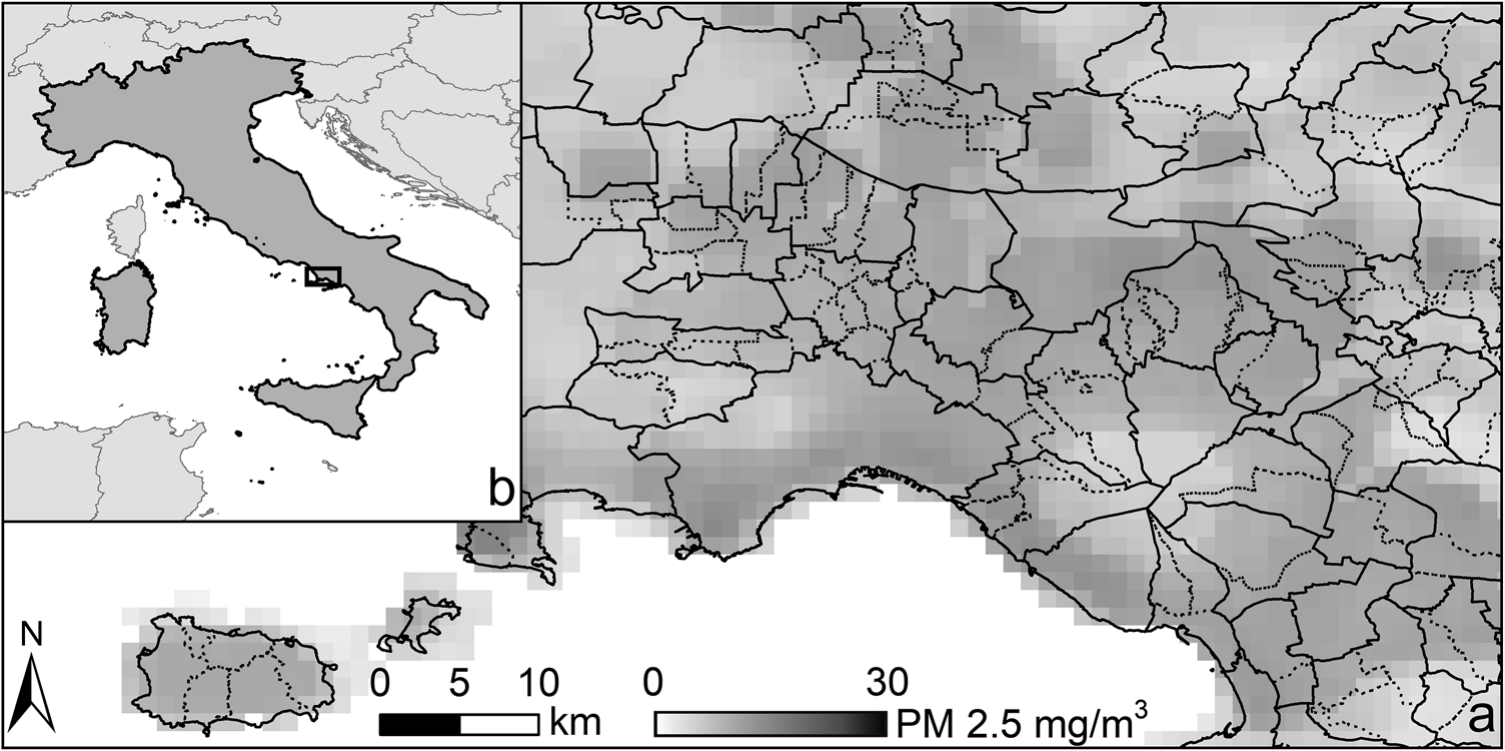
PM2.5 raster layer summarization by Italian communes. (**a**) A detail in the area of Naples allows to observe the impact of ‘smart dissolving’ small communes: the raster grid (grey scale) represents the PM2.5 concentration for each 0.01 degree pixels. The solid line is the results of dissolving the Italian communes using the proposed aggregation approach and by imposing an MMU of 16 km^2^. Dashed line shows the original communes boundaries. (**b**) The location (rectangle) of the area represented with (**a**) in the Italian peninsula. Map produced by the authors using ArcMap (Esri) 10.8 software.

According to the type of analysis to be conducted (e.g. a trade-off between precision in representation area and granularity of the municipalities PM2.5 data) the user can impose the most appropriate MMU. In our analysis, we selected the scenario ‘SD16’ as a reasonable compromise, with a significant reduction of the raster representation differences in area. This layer has 5583 polygons, with a mean area of 53.99 km^2^ and seven municipalities remaining below the MMU (isolated islands). On average, municipalities are merged with other two municipalities and the maximum aggregation is found in an area close to Asti (central Piedmont Region) where 19 municipalities are dissolved together reaching an area of 140 km^2^. Figure 3 shows the result of the scenario ‘SD16’ by comparing the dissolved layers with the original Italian municipalities’ layer and the raster grid of PM2.5 to be summarized, in the region of Naples.

Overall results from the zonal statistics (Supplementary Material S1) indicate the dissolved municipalities of Badia Pavese and Pieve Porto Morone, in the southern part of Lombardy, as the area with the highest mean concentration of PM2.5 in 2011 (22.36 mg/m^3^). These municipalities are followed by the municipality of San Giovanni Lupatoto (22.05 mg/m^3^), in Verona’s outskirts; Cavallino-Treporti (21.91 mg/m^3^), on the eastern side of Venice’s Lagoon, and Travacò Siccomario, south of Pavia (21.70 mg/m^3^). The first 20 entries (representing 31 municipalities) are mostly located in ‘Pianura Padana’ along the axes Milan-Brescia, one of the most industrialized areas of Italy^33^. However, the highest peaks of concentration (i.e. maximum pixel value) are found in the area of Venice’s Lagoon and Genova: the municipalities of Chioggia (25.20 mg/m^3^) and Cavallino-Treporti (24.70 mg/m^3^) followed by Genova (24.60 mg/m^3^) and Albissola (24.50 mg/m^3^) are the four highest in the chart. On the other hand, the ‘cleanest’ air, only considering PM2.5 concentration, is found in Sicily that occupies the top entries (> 100 municipalities): municipalities of Calamonaci (3. 578 mg/m^3^), Racalmuto (3.58 mg/m^3^) in Southeast of the island are the top two.

Moreover, the correlation analysis performed between PM2.5 concentration and population densities in resulting units shows a low correlation (r = 0.23, p << 0.001) with a low variance explained by the linear model (R^2^ = 0.05). This result does not reflect the strong correlation found by Wang et al*.*^31^ in China, but can be explained by the geographical and socioeconomic structure of the Italian peninsula. PM2.5 distribution shows a south to north gradient (from low concentration to high) that reflects the concentration of population and industrial activity in the northern ‘Pianura Padana’. This physiographic region has specific climatic features influenced by surrounding mountains, which form a barrier for the particulate matter dispersion by limiting the exchange of air with surrounding areas. Despite the scarcity of research on PM2.5 over the full Italian territory (studies are usually conducted for major cities due to lack of reliable spatiotemporal estimates of particle exposure in nonurban settings) these results are in line with previous findings of modelling efforts^13,34^.

### Estimation of boundaries of Metropolitan Areas in Portugal

The dataset of Urban Agglomerations with more than 300,000 inhabitants provided in the UN World Urbanization Prospects (WUP) has been one important reference data for urban analyses since its release. Updated every 4 years, it collects data at a global level about major urban areas of the world with a time series of their total population (from 1950) and a projected demography (until 2030). These data are used for urbanisation analyses and studies of demography in urban areas. Each entry (‘city’) of the dataset is associated with a position (latitude, longitude pairs) and it comprises, in its 2018 revision^35^, 1860 cities in the world. However, for many geospatial studies, knowing the mere point location of the city is not enough, and the polygonal boundary of the city is a basic requirement. Among these studies, there are analyses of Sustainable Development Goals (e.g. SDG goal 11, especially indicator 11.3.1 Land Use Efficiency); comparison of city extent and density; evaluation of exposure to hazards in urban areas; and many others.

In the following application, we propose a methodology to delineate the boundaries of the WUP Urban Agglomerations (WUP table F22; https://population.un.org/wup/Download/) by means of the proposed aggregation algorithm based only on the point location and reported total population of the city, and using a layer of administrative units (e.g. municipalities) as building blocks of the aggregation. As a proof-of-concept of the methodology we applied this to the Portuguese Metropolitan Areas of Lisbon and Porto because the actual boundaries of these two Metro Areas are clearly defined and known, as being administrative entities composed of municipalities (thus enabling to validate approach). The approach can be easily extended to the complete WUP dataset, but would lack a reference for validation. Therefore, for the purpose of showing the approach and validating it, we limit the exercise to the two mentioned Metro Areas.

We retrieved the boundaries of the census sections of mainland Portugal from the open 2011 national Population and Housing Census^36^ and we aggregated units to the municipal level. The obtained layer contains 278 municipalities with their code (‘DTCC01’), the resident population of 2011 (‘RPOP11’), and the encoding of the upper administrative level (‘NUTS3’). The historical reference population for Portuguese municipalities from 1961 to 2011 (by decade) was also produced by INE and retrieved from Eurostat. Mainland Portugal’s municipalities in 2011 have a mean area of 320 km^2^ (ranging from 8 km^2^ of São João da Madeira to 1724 km^2^ of Odemira) and a mean 2011 population of 36,142 people per municipality (ranging from 1834 people of Barrancos to 547,693 people of Lisbon).

We set the exercise as a MAV problem by using the ‘Seeds’ ordering algorithm: using the ‘Selection by Location’ ArcGIS tool, first we identified the two polygons corresponding to the WUP locations of Lisbon and Porto, the seat and main city/municipality of respective Metro Areas. We set these two municipalities as Seeds for the respective Metro areas and impose for each one the respective population of Urban Agglomeration as reported in the WUP dataset (Table 2). In order to accommodate possible small discrepancies between WUP reported population and actual resident population as compiled in the 2011 national census, we set as target population 95% of the value in WUP dataset, introducing a 5% tolerance. Therefore, the algorithm will merge polygons adjacent to each seed municipality by summing the ‘Target Field’ ‘RPOP11’ until it reaches the imposed thresholds. We defined the ‘Dissolve Rule’ to ‘MaxDensity’ as we assume that municipalities belonging to the Metropolitan Area share higher population densities than others. Given that Metropolitan Areas are often hierarchically nested into other administrative levels, we also imposed that polygon adjacency should only be considered in first instance within the upper administrative level, by imposing the ‘Bound Adjacency Field’ parameter, set to ‘NUTS3’, and only in a later stage (i.e. if the aggregation of all polygons within the NUTS3 does not reach the target) among the rest of the polygons.

**Figure 4:**
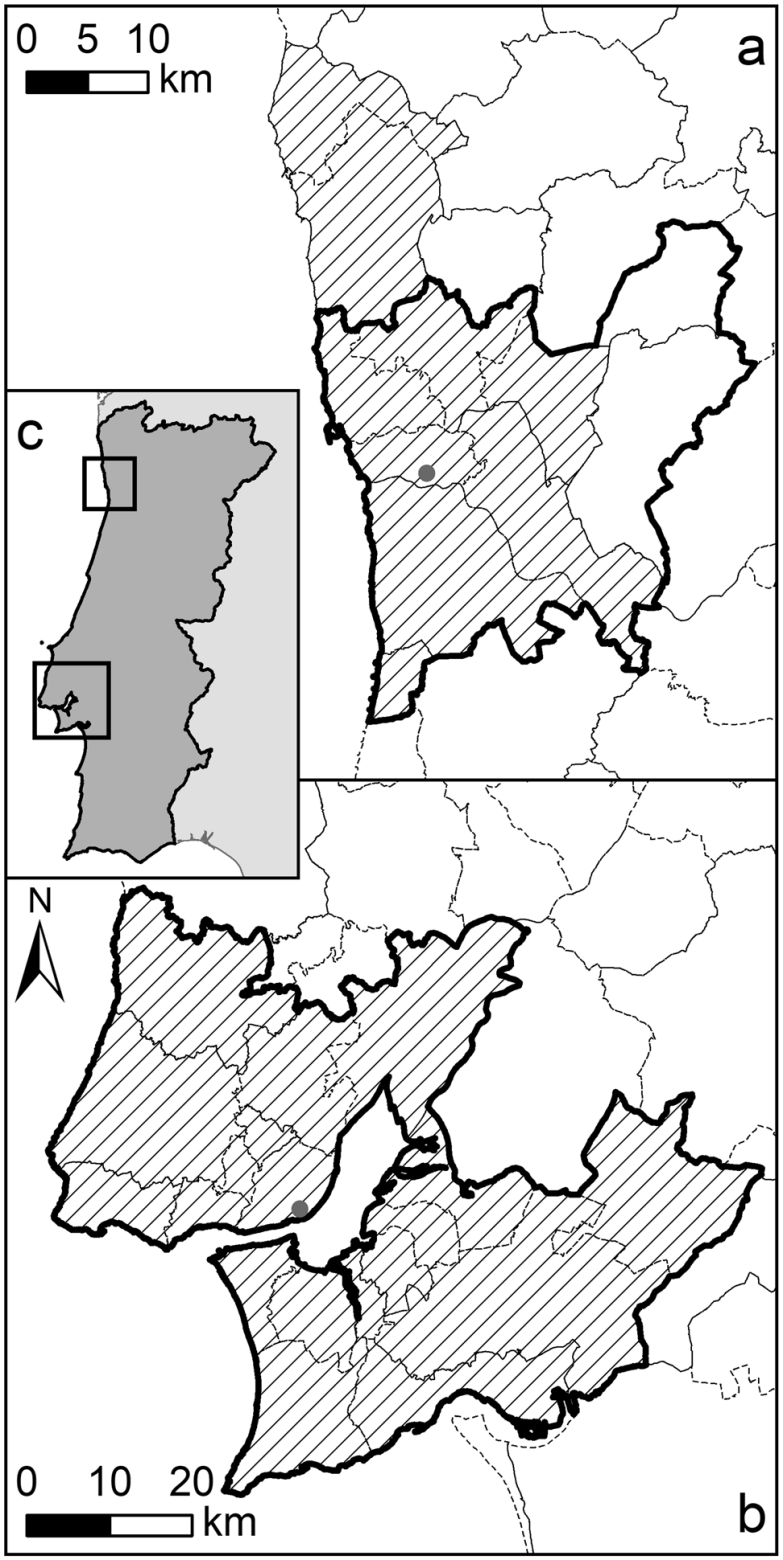
Estimation of Metropolitan Area boundaries for Porto (**a**) and Lisbon (**b**). Dashed lines represent the municipality boundaries; grey points are the locations of each urban agglomeration provided by WUP dataset; the solid black lines show the estimated boundaries by means of ‘GHS-SmartDissolve’ tool, imposing as target for polygon aggregation the reaching of 95% of WUP 2011 population (i.e. 5% tolerance to accommodate eventual small discrepancies between WUP and census data); striped areas are the actual Metropolitan Areas of Porto and Lisbon in 2011. (**c**) The locations of the areas represented with (**a**) Porto (upper rectangle) and (**b**) Lisbon (lower rectangle) in Portugal mainland. Map produced by the authors using ArcMap (Esri) 10.8 software.

Figure 4 shows the results of the exercise comparing the estimated boundaries of Metropolitan Areas with their actual boundaries. The estimation for Lisbon Metro (Fig. 4b) shows a perfect spatial match between the estimate and the real extent. The total 2011 population reported in Lisbon Metropolitan Area by WUP is 2,825,780 people while the 18 aggregated municipalities reach 2,821,838 people, a 99.9% match. The estimated boundary for Porto Metropolitan Area in 2011 (Fig. 4a) accounts for nine aggregated municipalities, as the actual Metropolitan Area then, with a total population match of 99.9%: 1,287,360 people in the estimated boundaries versus 1,288,020 reported in the WUP dataset. However, the spatial comparison shows a mismatch in the eastern and northern borders with two municipalities, Paredes and Paços de Ferreira, which are dissolved with the main Porto area instead of the two northern municipalities, Vila do Conde and Póvoa de Varzim. This mismatch is due to historical factors that link the two latter municipalities to Porto. These two municipalities have been historically connected to the city of Porto by a railway line, which enabled establishment of commuting flows. Unfortunately, the numerical algorithm that found higher densities and coherent population values in the municipalities of Paredes and Paços de Ferreira in 2011, cannot reproduce such historical factors. Still, the automated procedure developed produces an overall match of 93% of municipalities (25 over 27) for the two Metropolitan Areas that can be considered a quite positive result.

## Discussion and conclusions

Aggregating polygons that store count data is not a trivial procedure, in particular when a user requires more control over the dissolution process than that offered by standard geospatial tools. For example, when the problem requires to reach a specific MMU/MAV the procedure is particularly challenging as it can be performed only through manual geoprocessing.

To meet such needs of flexibility in an automated way, we develop an approach that suits advanced spatial analysis and required aggregation of vector features while preserving the native integrity of count values, both in the output aggregated feature and in the study area (volume). The wide set of available parameters allows the user to customize each analysis according to the requirements and details of the study. The main strengths of this method are the use of area threshold or field value threshold to be targeted by the dissolution, with the possibility of selecting different dissolve rules and of setting specific polygon analysis ordering, while ensuring the correct handling of multipart polygons and preserving the input geometry (i.e. no generalization of outlines). As shown in the first example application, this tool can mitigate the mismatch in scales between two layers that need to be combined to perform a required analysis. These capabilities can also mitigate the infamous Modifiable Areal Unit Problem (MAUP^18^), by generating layers with a more uniform distribution of the feature area at a different scale (aggregation) and geometry from the one used to report the phenomenon, allowing the comparison of results among the different aggregation levels. By mitigating the impact of MAUP the tool can also benefit data exploration activities, e.g. by enabling aggregation of features to a target MMU before conducting correlation or regression analyses^37^.

In the specific context of modelling and analysis involving population, this tool can contribute at least in three ways by enabling consistent aggregation of population zones: (i) model exploration and testing of hypotheses, including exploratory analysis of association of population distribution with covariates (e.g. built-up area); (ii) aggregation of population source zones used in population gridding—in absence or regardless of an explicit hierarchy of administrative levels (e.g. municipalities, districts, provinces, etc.); and (iii) aggregation of spatial units used for training population distribution models and validation of output raster grids.

The flexibility of the approach also allows testing and comparing different parameter settings and selecting the best performing scenario for the purposes of the analysis. The capabilities of the proposed aggregation algorithm were illustrated with two types of real-world applications, one common in environmental analyses, involving reaching an MMU suitable for spatial analysis, and another aiming at attaining a given MAV. In the application of the approach to summarize PM2.5 concentration data in Italian municipalities, we chose to perform the analysis by obtaining the most compact polygon possible, but other options are possible. For example by using the ‘MinValue’ dissolution rule pointed to area, municipalities would be dissolved with the smallest adjacent one, potentially avoiding that the aggregated PM2.5 values would be biased towards the value of much larger polygons. Another possibility is to limit aggregation to polygons belonging to the same province or region, in that way preserving the hierarchical administrative structure and enabling analysis of results by those administrative levels. In this application, we were able to associate to all municipalities (or aggregation of municipalities) a mean annual value of PM2.5 and show the absence of a correlation with population density while highlighting the strong north–south gradient of particulate matter concentration.

The modelling approach implemented is not exempt from criticism and limitations. Notwithstanding our efforts to implement the most complete aggregation rules and constraints, we showed in the Metropolitan Areas application that the result provided by the approach reduces the need for manual identification of polygons to be dissolved, but it is subject to mis-assumptions in boundary estimation. Despite these limitations, we are confident that our software can be valuable in extending this approach to all 1860 WUP Urban Agglomerations in the world with minimal issues. Moreover, the tool allows to develop more refined estimations using ‘Target Upper Bound’ and ‘Minimum Perimeter Share’ parameters, or exploiting other attribute fields collected in ad hoc analyses, to improve the estimates. A downside to the flexibility of the model is the sensitivity to the provided ordering of the seed identifiers (only in ‘Seeds’ ‘Ordering rule’) or the polygon order in the shapefile (in the ‘ID’ ‘Ordering rule’) that could produce, in some particular cases (i.e. seed polygons extremely close together in the first case, or polygons below the target threshold in the second), slightly different results. However, the solution for this would have excessively increased the computational time by computing all possible results for any seeds ordering. Another limitation is related to the necessity of having no topological issues along polygon borders, as the tool is not able to compute the length of the shared border and therefore may produce imprecise results when perimeter length is involved.

Differently from similar commercial software, the ‘GHS-SmartDissolve’ tool implementing the proposed approach is provided as a free toolbox suitable for ArcGIS (Esri) and standalone application, with foressen developments to include new dissolve rules, other multi-criteria methods when multiple dissolve rules are selected, and more formulas for updating fields. The implementation of the core algorithm in MATLAB^®^ code, allows 64-bit and multiprocessing computing, and requires the installation of the free MATLAB^®^ Compiler Runtime (MCR), automatically installed within the installation wizard of the tool.

‘GHS-SmartDissolve’ tool is freely available for non-commercial, research and educational uses through the ‘Tools’ page on the Global Human Settlement Layer (GHSL) website (http://ghsl.jrc.ec.europa.eu/). We intend to maintain and further develop the ‘GHS-SmartDissolve’ tool for responding to users’ needs and suggestions. Beside extending some functionalities already implemented, potential updates would include (i) the possibility to combine MMU and MAV problems in a single run; (ii) the dissolution rule ‘affinity’ to aggregate only similar polygons, according to a priority table (defined by the user, or included in the attribute fields), and the ‘strict affinity’, that would avoid aggregating dissimilar polygons. Future releases will be available in the same repository.

## Supplementary Information

Supplementary Information.

## Data Availability

GHS-SmartDissolve tool: toolbox freely available for download in the GHSL (EC-JRC) repository: https://ghsl.jrc.ec.europa.eu/tools/GHS-SmartDissolve_arcgis_toolbox_installer.exe. Particulate matter exercise: Global Annual PM2.5 Grids (2011) are available in the SEDAC (NASA) repository upon user registration: https://sedac.ciesin.columbia.edu/downloads/data/sdei/sdei-global-annual-gwr-pm2-5-modis-misr-seawifs-aod/sdei-global-annual-gwr-pm2-5-modis-misr-seawifs-aod-2011-geotiff.zip. Italian communes 2011 boundaries are available in the ISTAT repository: https://www.istat.it/storage/cartografia/confini_amministrativi/non_generalizzati/Limiti2011.zip. Italian communes 2011 population are available in the ISTAT repository: http://dati-censimentopopolazione.istat.it/Index.aspx?lang=en. Selected table: ‘Population’ —> ‘Resident population and present population’ —> ‘Absolute values’ —> ‘Municipalities’ —> ‘Present population by gender’. Estimation of Metropolitan Areas boundaries exercise: Urban Agglomeration data of the 2018 World Urbanization Prospects is available in the UNDESA repository: https://population.un.org/wup/Download/Files/WUP2018-F22-Cities_Over_300K_Annual.xls. Census 2011 section boundaries in Portugal are available in the INE repository: https://www.ine.es/censos2011_datos/cartografia_censo2011_nacional.zip. Historical population of municipalities in Portugal, 1961–2011, is available in the EUROSTAT repository: https://ec.europa.eu/eurostat/documents/345175/6787248/Population_data.zip. All datasets used and/or analysed during the current study available from the corresponding author on reasonable request.

## Abbreviations

GHS: Global Human Settlement
GHSL: Global Human Settlement Layer
GIS: Geographic Information System
GIScience: Geographic Information Science
INE: Instituto Nacional de Estatística
ISTAT: Istituto Nazionale di Statistica
MAUP: Modifiable areal unit problem
MAV: Minimum attribute value
MCA: Maximum combined area cell assignment in rasterization procedure
MCR: MATLAB compiler runtime
MMU: Minimum Mapping Unit
UNDESA: United Nations Department of Economic and Social Affairs
WUP: World Urbanization Prospects
